# Editorial: Motivations for physical activity

**DOI:** 10.3389/fpsyg.2024.1437220

**Published:** 2024-06-21

**Authors:** Aleksandra M. Rogowska, Pedro Morouço

**Affiliations:** ^1^Institute of Psychology, University of Opole, Opole, Poland; ^2^ESECS, Polytechnic University of Leiria, Leiria, Portugal; ^3^CIDESD, Research Center in Sports Sciences, Health Sciences and Human Development, Vila Real, Portugal

**Keywords:** basic psychological needs (BPN), motivation to physical activity (PA), motivation to sports training, physical activity intervention, Self-Determination Theory (SDT), self-efficacy, Theory of Planned Behavior (TPB), Health Action Process Approach (HAPA)

## Introduction

Engaging in physical activity is a complex and multifaceted phenomenon driven by a variety of motivations. This Research Topic sought to understand the factors that inspire individuals to participate in physical activities (PA). The objective of this Research Topic of articles is to provide fresh perspectives on this subject, thereby offering innovative insights into motivational factors, which is an incredibly promising and relevant domain, especially considering the growing importance of physical activity for health and wellbeing around the world. Addressing the complexity of the motivations behind physical activity can lead to a deeper understanding of exercise-related behaviors and, in turn, inform more effective strategies for promoting active lifestyles.

Including a wide range of target populations, such as college students, older adults, and people affected by the COVID-19 pandemic, is crucial to ensure that the findings have relevance and applicability in diverse contexts. In addition, consideration of social, cultural, and environmental factors reflects the interdisciplinary and multifaceted nature of physical activity and human behavior in general. With this Research Topic, we hope to offer valuable insights into the complex domain of motivations for physical activity and underline the importance of individual characteristics, social and cultural factors, and environmental influences in shaping exercise behaviors. Our goal was also to highlight research findings that have implications for the promotion of physical activity in diverse populations, including college students, older adults, and those affected by the COVID-19 pandemic. Three areas of motivation for PA have been identified:

## Factors increasing motivation to engage in physical activity

An integrated theoretical perspective that blended the Health Action Process Approach (HAPA) and the Theory of Planned Behavior (TPB) was applied in a cross-sectional study conducted by Meng et al. to investigate psychosocial factors determining the initiation and maintenance of physical exercise behaviors in Chinese individuals with substance use disorders (SUD). An extensive set of variables was explored, including task self-efficacy (TSE), maintenance self-efficacy (MSE), recovery self-efficacy (RSE), outcome expectations (OE), action planning (AP), coping planning (CP), social support (SS), subjective norms (SN), attitude behavior (AB), behavioral intention (BI), perceived behavioral control (PBC), and risk perception (RP). The structural model revealed several associations that provide valuable scientific guidance for improving physical exercise adherence among individuals with SUD.

Motivational differences between men and women and among individuals with varying relationship statuses were investigated in a study focused on determining the most effective exercise program among three types of social interaction: people exercising in fitness alone, in aerobic groups, and with a personal trainer (Vuckovic and Duric). The findings indicated that individuals who exercised alone were primarily motivated by intrinsic factors, such as enjoyment and stress management. On the other hand, health-related motives, such as avoiding ill health, were most commonly associated with exercising with a personal trainer, particularly among females and those in a relationship.

The study conducted by Gabay et al. aimed to investigate the contextual variables and factors that influence adherence to physical activity among veteran, novice, and dropout trainees in Israel. The findings suggest that individuals with varying ages, gender, and levels of physical training experience have unique and diverse goals for their training, and there is no single goal that is suitable for everyone. By identifying the most critical factors that may impact adherence to physical activity, self-efficacy can be increased simultaneously.

A cross-sectional study investigated the part played by physicians in encouraging physical activity among pregnant women in Poland (Laudańska-Krzemińska and Krzysztoszek). According to the WHO recommendations for physical activity, 41% of women adhered to these guidelines before becoming pregnant. Among pregnant women who were physically inactive, poorer wellbeing was reported. Regrettably, healthcare professionals do not often provide education and motivation to pregnant women regarding physical activity.

A systematic review (Sun et al.) was conducted employing a socio-ecological framework to identify factors that serve as either facilitators or obstacles to engaging in physical activity (PA) among pregnant women. The study revealed that higher levels of education, knowledge, and skills, as well as access to mass media, had a positive influence on PA in pregnant women. Conversely, lower levels of education, inadequate knowledge and skills, low income, pregnancy discomforts, limited time, safety concerns, and societal perceptions of PA during pregnancy were identified as significant barriers. Furthermore, significant others, including family members, colleagues, friends, and partners, can either support or hinder PA. It is essential to offer accessible information and resource systems to pregnant women to foster a healthy lifestyle during pregnancy. Additionally, interventions should aim not only at pregnant women but also their families.

## Motivation to participate in different sport disciplines

The prospective study by Honório et al. investigated the anthropometric characteristics and other relevant variables associated with physical performance in a sample of Portuguese U-17 soccer players during 10 months of soccer training. Results indicated significant enhancements in the levels of anthropometric and physical fitness variables, such as leg power, speed, bone mass, muscle mass, and fat mass. These findings suggest that soccer is a multifaceted collective sport that fosters the development of various capacities, including power, agility, joint flexibility, and muscle development, in a harmonious manner.

The impact of coach-delivered verbal encouragement on the physiological and psychological responses was investigated in four sessions of small-sided games (SSGs) among male basketball players from Tunisia (Khayati et al.). The results indicated significant benefits of coach-delivered verbal encouragement on both the physical and psychophysiological responses of adolescent athletes, including increased physical enjoyment, positive mood state, lower heart rate, and higher physical activity intensity level. Therefore, it is recommended that coaches incorporate verbal encouragement strategies to enhance sports performance.

The study by Leduc et al. tested basic psychological need satisfaction among Canadian collegiate athletes, focusing on their interactions with team identification and leader-member exchange (LMX) perceived quality. The results demonstrated a positive association between team identification and the satisfaction of the needs for competence and relatedness. Additionally, satisfaction with the needs for competence and autonomy was shown to be positively related to LMX quality. Furthermore, LMX quality was found to moderate the relationship between team identification and the satisfaction of the needs for competence and relatedness, emphasizing the significant role played by team identification and LMX quality in satisfying the basic psychological needs of collegiate athletes.

The international study of motivational drivers and a sense of belonging among Chinese Martial Arts (CMAs) practitioners was conducted using the Self-Determination Theory (SDT) by Cao and Lyu. The motivation for practicing CMAs was found to consist of enjoyment, mastery, physical condition, psychological condition, and appearance. The persistence in practicing CMAs was positively related to motivation for practicing CMAs, sense of belonging, affiliation, competition ego, and others' expectations. International instructors of CMAs should focus on fostering the development of physical, mental, aesthetic, and moral qualities of CMAs practitioners, together with virtues and etiquette. By doing so, instructors can help practitioners discover enjoyment in their practice, achieve competency in their skills, and ultimately enhance their overall performance.

The motivation for participating in Brazilian Jiu-Jitsu (BJJ) was examined in accordance with the Self-Determination Theory (Tarver and Levy). The primary motives that emerged were interest/enjoyment, competence, and fitness, while appearance and social motivations were found to be less pronounced in BBJ players from the USA. It was observed that competence and interest/enjoyment motivators had a significant impact on competitive BJJ players, regardless of their years of experience in the sport. The results of this study could be helpful for coaches, sports clinicians, and sports psychologists in developing training programs that are tailored to the motivations of BJJ players.

## A novel approach for assessing and applying motivation to physical activity

A mixed-methods study was performed in Norway to evaluate the adoption, acceptability, and sustained use of digital interventions to encourage physical activity among inactive adults (Manskow et al.). The participants in the randomized controlled trial received a wearable activity tracker along with the personalized metric Personal Activity Intelligence (PAI) on a mobile app. Furthermore, two groups received access to online training, and one group also had access to online social support. The study results indicated that PAI was the most effective intervention, with satisfactory usability and positive effects on motivation and behavior change, leading to high adoption and sustained use.

A four-month randomized controlled trial was carried out among healthy adults in Singapore to investigate the effectiveness of hedonic vs. cash incentives in promoting physical activity (Finkelstein et al.). The study revealed that both the cash and hedonic incentive groups showed an increase in mean daily steps without any significant differences. These results imply that either type of incentive can be implemented to increase physical activity among inactive adults.

The Behavioral Regulation in Exercise Questionnaire (BREQ-3) was validated in a sample of German-speaking young adults (Cocca et al.). The 22-item BREQ-3 questionnaire, which encompasses six factors, demonstrated good fit and moderate to high internal consistency in the German-speaking population. Additionally, the BREQ-3 proved to be invariant across genders. Consequently, the BREQ-3 can be considered a scientifically valid instrument for assessing certain social aspects of exercise behaviors in future studies.

We hope that this Research Topic of articles will result in new insights that can inform policies, programs, and interventions aimed at promoting physical activity and ultimately contribute to the improvement of the health and quality of life of individuals around the world.

## Author contributions

AR: Writing – review & editing, Writing – original draft, Conceptualization. PM: Writing – review & editing, Writing – original draft, Conceptualization.

